# The CD3-Zeta Chimeric Antigen Receptor Overcomes TCR Hypo-Responsiveness of Human Terminal Late-Stage T Cells

**DOI:** 10.1371/journal.pone.0030713

**Published:** 2012-01-23

**Authors:** Gunter Rappl, Tobias Riet, Sabine Awerkiew, Annette Schmidt, Andreas A. Hombach, Herbert Pfister, Hinrich Abken

**Affiliations:** 1 Tumor Genetics, Dept. I Internal Medicine, University of Cologne, Cologne, Germany; 2 Institute for Virology, University of Cologne, Cologne, Germany; 3 Bundeswehr Institute of Pharmacology and Toxicology, Munich, Germany; 4 Center for Molecular Medicine Cologne, University of Cologne, Cologne, Germany; Carl-Gustav Carus Technical University-Dresden, Germany

## Abstract

Adoptive therapy of malignant diseases with tumor-specific cytotoxic T cells showed remarkable efficacy in recent trials. Repetitive T cell receptor (TCR) engagement of target antigen, however, inevitably ends up in hypo-responsive cells with terminally differentiated KLRG-1^+^ CD57^+^ CD7^−^ phenotype limiting their therapeutic efficacy. We here revealed that hypo-responsiveness of CMV-specific late-stage CD8^+^ T cells is due to reduced TCR synapse formation compared to younger cells. Membrane anchoring of TCR components contributes to T cell hypo-responsiveness since dislocation of galectin-3 from the synapse by swainsonine restored both TCR synapse formation and T cell response. Transgenic expression of a CD3-zeta signaling chimeric antigen receptor (CAR) recovered hypo-responsive T cells to full effector functions indicating that the defect is restricted to TCR membrane components while synapse formation of the transgenic CAR was not blocked. CAR engineered late-stage T cells released cytokines and mediated redirected cytotoxicity as efficiently as younger effector T cells. Our data provide a rationale for TCR independent, CAR mediated activation in the adoptive cell therapy to avoid hypo-responsiveness of late-stage T cells upon repetitive antigen encounter.

## Introduction

Adoptive T cell therapy recently showed significant success in the treatment of malignant diseases [1], [2]. Upon adoptive transfer tumor-specific T cells migrate to the tumor tissue, engage antigen and mediate a pro-inflammatory anti-tumor response. At the molecular level, the TCR components form an immunological synapse at the interface of the effector T cell to the peptide-MHC complexes of the antigen-presenting cell to initiate T cell activation by downstream signaling [3]. Lipid rafts in the membrane substantially impact this process and the distribution and the amount of lipid rafts substantially changes during T cell differentiation. In particular there are clear differences in the lipid raft distribution and TCR synapse formation between naïve and effector memory cells; naïve T cells have fewer rafts in their plasma membrane and require CD28 costimulation to amplify TCR signaling whereas effector memory T cells have more rafts and the signal amplifies in the absence of costimulation [3], [4]. At the moment several companies are developing drugs targeting molecules at the immunological synapse. Some biochemical substances or monoclonal antibodies are currently tested in clinical studies to improve the efficacy of immunological synapse formation for cancer immunotherapy [5]. Success in these studies was already reported, since ipilimumab, a monoclonal antibody against CTLA-4, produces durable, complete responses in a small but consistent proportion of melanoma patients [5].

With respect to the therapeutic efficacy central memory T_(CM)_ showed superior over effector memory T(_EM_) cells by proliferating more rapidly upon antigen encounter and by persisting longer upon adoptive transfer, thereby promoting repetitive migration into the draining lymph node and re-entering circulation [2], [6]. Repetitive antigen engagement, however, drives T cells to progress in the differentiation pathway inevitably ending up in terminally differentiated cells with the KLRG-1^+^ CD45RO^bright^ CD57^+^ CD7^−^ late-stage phenotype [7], [8]. T cells in this stage show a high activation threshold, a high propensity to undergo activation induced cell death (AICD) [9] and a hypo-responsiveness upon TCR stimuli [10]. A hypo-responsive TCR, however, limits the therapeutic efficacy of adoptive T cell therapy [11].

To engraft adoptive T cell therapy with defined specificity, patient's T cells are *ex vivo* engineered with a transgenic TCR or alternatively with a chimeric antigen receptor (CAR) that in contrast to the TCR consists of a single polypeptide chain with an extracellular antibody-derived binding domain and the intracellular CD3-zeta signaling moiety [12]. While a transgenic TCR with CD3-zeta signaling domain forms synapses and initiates downstream signaling and effector functions independently of the endogenous TCR/CD3 complex [13], the functional activity of a transgenic CAR depends on the interaction with the signaling components of the endogenous TCR complex in the Jurkat T cell model [14]. No data, however, are so far available for engineered peripheral blood T cells, in particular for those T cells that experienced repetitive antigen engagement. The issue is considered critical for the success of clinical T cell therapy in order to control tumor growth by adoptively transferred T cells in long-term.

We here elucidated that the hypo-responsiveness of the physiological TCR in terminally differentiated, late-stage T cells is due to membrane anchoring of TCR components whereas the down-stream signaling pathway is still functional. Consequently, late-stage T cells are susceptible to CAR CD3-zeta-mediated activation resulting in cytokine release and redirected cytotoxicity despite hypo-responsive TCR. Our data provide a rationale for CAR redirected T cell activation in the adoptive immunotherapy to avoid hypo-responsiveness upon repetitive antigen encounter.

## Results

### Late-stage T cells are hypo-responsive to TCR stimulation

To record TCR mediated activation of terminally differentiated T cell subsets we isolated CMV-specific CD8^+^ T cells from CMV infected, HIV^−^, HLA-A2^+^ individuals. CMV-specific T cells, identified by pp65 tetramer binding, predominantly consist of late-stage KLRG-1^+^ CD57^+^ CD7^−^ T cells and a minor subset of intermediate-stage KLRG-1^−/low^ CD57^−^ CD7^+^ cells (Fig. S1). Cells of both subsets expressed TCR-alpha/beta and CD3-epsilon at similar levels and equally bound pp65 CMV tetramers indicating similar amounts of CMV-specific TCR molecules on their cell surface (Fig. S1).

**Figure 1:**
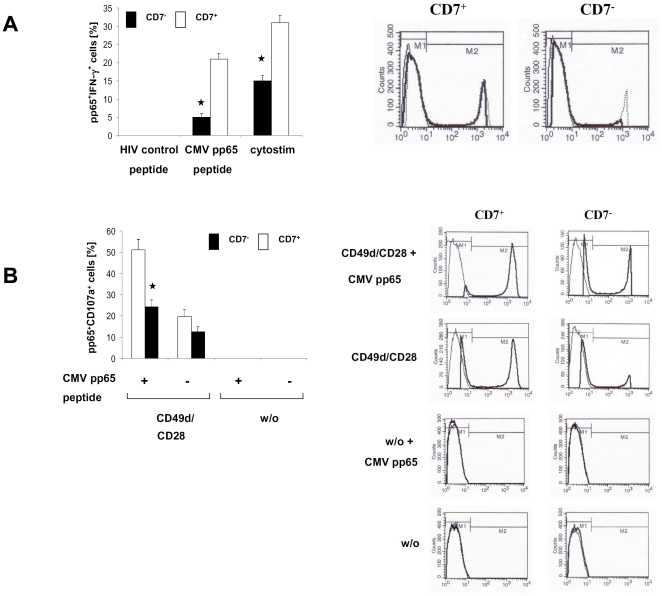
CMV-specific, late-stage T cells are hypo-responsive upon TCR stimulation compared to intermediate-stage T cells. (A) Freshly isolated PBMC's from CMV patients were activated by incubation with CMV pp65 peptide or for control with the HIV peptide or Cytostim, respectively, for 6 hrs. CMV-specific cells were identified by staining them with the PE-conjugated MHC tetrameric complexes and mAbs specific to CD8, CD7, CD45RO and subsequently recorded for IFN-gamma production by staining the with anti-human IFN-gammamAb. The number of tetramer-positive cells of the CD7^−^ and CD7^+^ cells of the CD8^+^ CD45RO^+^ subset with and without IFN-gammaproduction was recorded by flow cytometry. Histograms of one representative donor from five CMV patients is shown. Overlays of control peptide stimulated (thin line), Cytostim-activated (dotted line), and CMV peptide-stimulated (bold line) cells are shown. (B) CD107a was monitored by flow cytometry of CMV-specific CD8^+^ CD45RO^+^ T cells of the CD7^−^ and CD7^+^ subsets after or without CD49d/CD28 stimulation in presence or absence of CMV peptide. Histograms of one representative donor from five CMV patients is shown (CD107a: bold line; isotype control: thin line). Data in Fig. 1 A & B represent mean values ± standard error of the mean (SEM) of five donors and were compared using a paired t-test. * p<0.05.

CMV pp65-driven T cell activation, indicated by increase in IFN-gamma expression, was substantially reduced in T cells of the CD7^−^ late-stage subset compared to CD7^+^ intermediate-stage T cells (Fig. 1A). Activation was CMV-specific since HIV peptide as control did not induce IFN-gamma in those T cells. Antigen-independent TCR cross-linking moreover resulted in less activation of late-stage T cells compared to T cells in intermediate-stage of terminal differentiation. CD107a staining was reduced in late-stage T cells compared to intermediate-stage T cells upon pp65 CMV mediated activation indicating diminished cytolytic degranulation (Fig. 1B). As controls no increase in degranulation was monitored in CMV-specific T cell subsets when activated without CD49d/CD28 costimulation or in absence of antigen. Repression of T cell response is not restricted to CMV-specific T cells since polyclonal T cells in late-stage differentiation showed the same hypo-responsiveness after CD3 stimulation in presence of CD28 costimulation or IL-2 (Figs. S2A & B). Taken together we conclude that late-stage T cells are hypo-responsive in TCR mediated activation compared to intermediate-stage T cells.

### Late-stage T cells are deficient in TCR synapse formation

**Figure 2:**
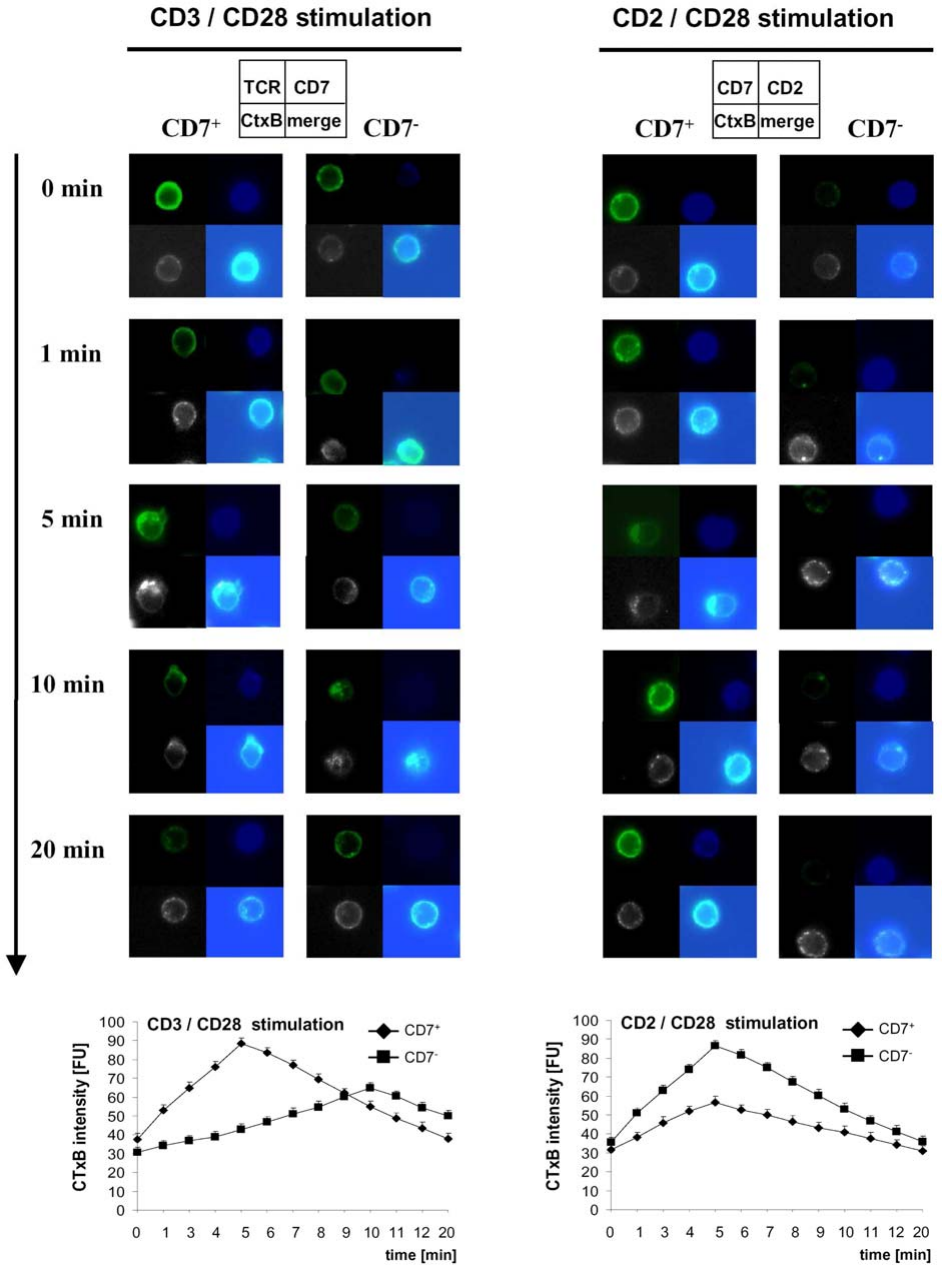
TCR synapse formation is impaired in late-stage T cells. CD8^+^ CD45RO^+^ T lymphocytes were incubated on coverslips coated with the agonistic anti-CD3 mAb (UCHT-1) plus anti-CD28 mAb (CD28.2) or with the anti-CD2 mAbs (L303.1) and (L304.1) plus the anti-CD28 mAb (CD28.2). Cells were activated for various time intervals (1 min until 20 min), incubation was stopped by addition of paraformaldehyde, and cells were stained with TCR-alpha/betamAb (green) plus CD7 mAb (blue) or alternatively with CD2 mAb (blue) plus CD7 mAb (green) together with cholera toxin B (CtxB) (white) for lipid raft staining. Cells were analyzed on a LSM 510 with 630× microscope magnification. A minimum of 100 cells for each data point was recorded. Representative images after 0 min, 1 min, 5 min, 10 min and 20 min stimulation out of five independent experiments are shown. Synapse formation intensity (CTxB intensity) was quantified as described in Materials and Methods. A minimum of 100 cells of each cell population on each coverslip was recorded. Data represent mean scores from five experiments ± standard error of the mean and were compared using a paired t-test. * p<0.05. FU: fluorescence unit.

We asked whether hypo-responsiveness of late-stage T cells is due to impaired TCR synapse formation. T cells were stimulated with the immobilized agonistic anti-CD3 antibody, alternatively with anti-CD2 antibodies, in presence of CD28 costimulation and TCR synapse formation was recorded by confocal microscopy. Within 5 min upon stimulation intermediate-stage T cells showed coalescence of TCR components into lipid rafts and TCR synapse formation in the contact region to antigen (Fig. 2). Late-stage T cells, in contrast, formed synapses with substantial delay, i.e., after 10 min, and with lower densities. No synapse formation was observed in T cells incubated on poly-*L*-lysine coated surfaces as control (data not shown). Both T cell subsets, however, recruited CD2 into lipid rafts within the same time upon CD2/CD28 stimulation indicating that impaired TCR synapse formation in late-stage T cells is restricted to TCR components and not due to an overall decrease in membrane fluidity.

**Figure 3:**
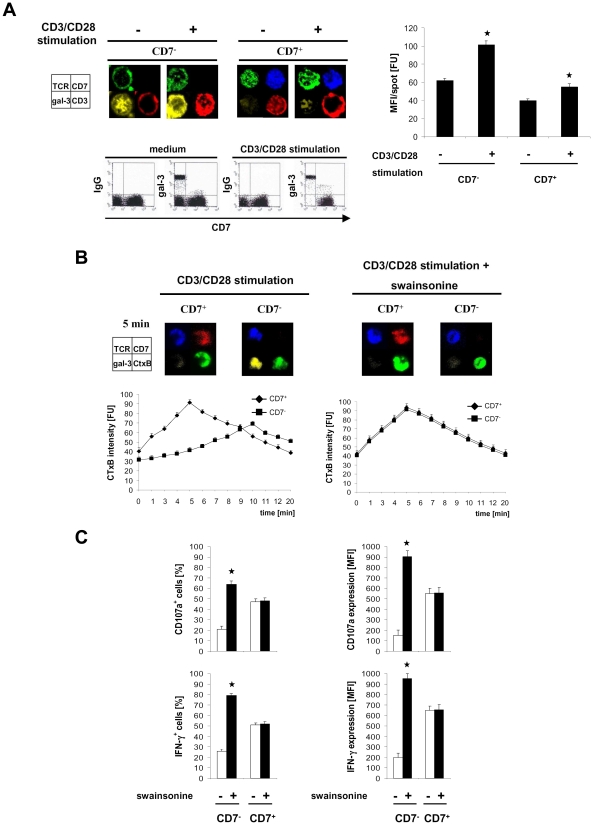
Galectin-3 prevents TCR synapse formation in late-stage T cells. (A) CD7^−^ and CD7^+^ T cells were activated by incubation with anti-CD3 mAb plus anti-human CD28 mAb or as control by an isotype-matched IgG1 (medium). Cells were stained for TCR-alpha/beta (green), CD7 (blue), CD3 (red) and for galectin-3 (yellow). Immunofluorescence was visualized by a LSM. Alternatively cells were stained with mAbs specific to CD7 and subsequently recorded for gal-3 expression by staining with the anti-human gal-3mAb or as control by an isotype-matched IgG and analyzed by flow cytometry. A representative experiment out of five is shown. Galectin-3 specific signals were quantitatively recorded by a LSM as described in Materials and Methods. A minimum of 100 cells for each data point was recorded. (B) To monitor location of galectin-3 in lipid raft formation, isolated CD7^−^ and CD7^+^ subsets of CD8^+^ CD45RO^+^ T cells were incubated with or without swainsonine for 24 hrs and then activated for various time intervals (1 min until 20 min) on coverslips coated with the agonistic anti-CD3 mAb plus anti-CD28 mAb. T cell stimulation was stopped with paraformaldehyde and cells were stained with anti-TCR-alpha/betamAb (blue), anti-CD7 mAb (red) and anti-galectin-3 mAb (yellow) together with CtxB (green) for lipid rafts staining. Immunofluorescence was visualized by a LSM and quantified as described in Materials and Methods. T cell staining after 5 min stimulation of one representative experiment out of five is exemplarily shown. (C) CD7^−^ and CD7^+^ subsets of CD8^+^ CD45RO^+^ T cells were incubated with or without swainsonine and activated as described in (B). Frequencies of cells producing IFN-gamma and showing degranulation indicated by CD107a was monitored by flow cytometry. Data in Fig. 3 represent the mean ± SEM of five experiments and were compared using a paired t-test. * p<0.05. FU: fluorescence unit; MFI: mean fluorescence intensity.

We asked whether impaired TCR synapse formation is due to reduced membrane mobility of TCR components as a consequence of galectin-3 binding to branched sugar residues as reported for other T cell subsets [15]. As shown in Fig. 3A, galectin-3 was located in the cytoplasm of resting late-stage T cells and translocated to the cell membrane upon CD3/CD28 stimulation where it co-localized with CD3-epsilon and TCR-alpha/beta. In contrast, intermediate-stage T cells showed no co-localization of galectin-3 with CD3-epsilon or TCR-alpha/beta. Galectin-3 was moreover expressed at higher levels and furthermore increased after CD3/CD28 activation in late-stage T cells compared to intermediate-stage T cells.

To revert galectin-3 block of TCR components, cells were treated with swainsonine that inhibits alpha-mannosidase II leading to impaired N-glycosylation and loss of branched sugar structures required for galectin-3 binding [15]. After treatment of late-stage T cells with swainsonine, galectin-3 no longer translocated to the cell membrane. TCR synapse formation upon antigen engagement occurred with similar efficiency and time frame in swansonine treated late-stage T cells as in intermediate-stage T cells (Fig. 3B). Treated late-stage T cells accordingly showed improved activation with respect to the secreted IFN-gamma levels, the number of IFN-gamma^+^ cells and the cytolytic degranulation of cells indicated by CD107a staining (Fig. 3C). Data indicate that impaired TCR synapse formation of late-stage T cells can be restored by preventing galectin-3 mediated membrane anchoring of TCR components.

### Transgenic expression of a CD3-zeta signaling CAR overcomes hypo-responsiveness of late-stage T cells

**Figure 4:**
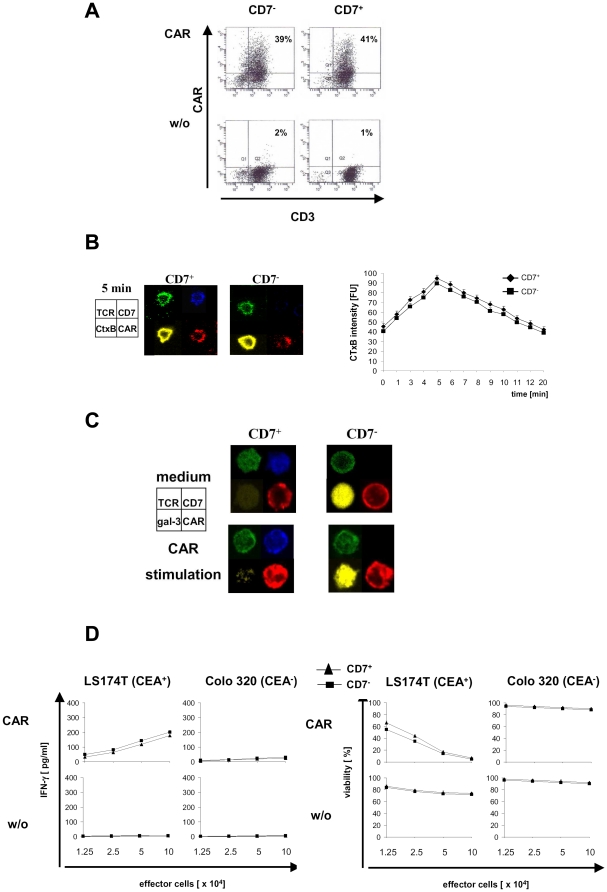
CAR triggered activation is as efficient in late-stage as in intermediate-stage T cells. (A) Isolated CD7^−^ and CD7^+^ subset cells of CD8^+^ CD45RO^+^ T cells were engineered with the CEA-specific CAR BW431/26scFv-Fc-CD3-zeta by retroviral gene transfer. CAR expression was monitored by flow cytometry with a PE-conjugated anti-IgG1 mAb and a FITC-conjugated anti-CD3 mAb. Dot plots of one representative transduction of CD7^−^ and CD7^+^ T cells from five independent experiments is shown (B) CAR modified T cells were activated for various time intervals (1 min until 20 min) on coverslips coated with the BW2064/36 mAb. The incubation was stopped with paraformaldehyde and cells were stained for TCR (green), CD7 (blue), CAR (red) and CtxB (yellow). Imaging after 5 min of activation is exemplarily shown. Synapse formation intensity (CTxB intensity) was quantified as described in Materials and Methods. (C) To monitor location of galectin-3 upon CAR-mediated activation, engineered CD7^−^ and CD7^+^ T cells were activated by the BW2064/36 mAb or an isotype-matched control antibody of irrelevant specificity as described in (B). Cells were stained for TCR-alpha/beta(green), CD7 (blue), CAR (red) and for galectin-3 (yellow). Imaging after 5 min of T cell activation of one representative experiment out of five is exemplarily shown. (D) To monitor CAR-mediated effector functions, CAR engineered CD7^−^ and CD7^+^ T cells in increasing numbers were co-incubated with CEA^+^ LS174T and CEA^−^ Colo320 tumor cells, respectively. IFN-gamma in the culture supernatants after 48 hrs was monitored by ELISA. Viability of tumor cells was colorimetrically determined by XTT assay. Data in Fig. 4 represent the mean of five experiments ± SEM and were compared using a paired t-test. * p<0.05. FU: fluorescence unit.

Based on the assumption that hypo-responsiveness of late-stage T cells is exclusively due to impaired synapse formation without additional impairments, we hypothesized two fundamental consequences: (i) the TCR down-stream signaling pathway is functional and (ii) a CD3-zeta signaling CAR bypasses the TCR hypo-responsiveness redirecting late-stage T cells to a full anti-tumor cell response. We therefore engineered those T cells with a CAR with MHC-independent binding specificity for carcinoembryonic antigen (CEA) and the CD3-zeta signaling endodomain for T cell activation. Upon retroviral transduction T cells of the CD7^−^ late-stage and CD7^+^ intermediate-stage subset expressed the CAR with nearly same efficiencies (Fig. 4A). Life imaging revealed that late-stage T cells as efficiently formed CAR synapses as did intermediate-stage T cells upon CAR engagement of antigen. CAR synapse formation occurred with similar time kinetics in both T cell subsets (Fig. 4B). Galectin-3 remained in the cytoplasm upon antigen engagement without translocation to the cell surface (Fig. 4C) No co-localization of galectin-3 with the TCR occurred in those cells. Accordingly, CAR engagement of CEA^+^ target cells activated late-stage T cells equally efficiently as intermediate-stage T cells indicated by increase in IFN-gamma secretion and induction of redirected cytolysis (Fig. 4D). CAR-mediated T cell activation was antigen-triggered since co-incubation with CEA^−^ tumor cells did not induce IFN-gamma secretion or cytolysis. Incubation of non-modified T cells with tumor cells did not induce T cell activation. We conclude that TCR hypo-responsiveness of late-stage T cells can be circumvented by a transgenic CAR that triggers the TCR/CD3-zeta down-stream signaling pathway.

## Discussion

Repetitive antigen engagement of adoptively transferred T cells will inevitably end in terminally differentiated late-stage T cells [2], [16] which are functionally exhausted und dampen the therapeutic efficacy [10], [17], [18]. This phenomenon is of particular relevance when redirecting T cells with defined specificity towards target cells. While reduced response of early-stage CD45RO^+^ T cells is due to down-regulated expression of TCR components, especially the CD3-gamma chain, following ligand triggering [19], we here revealed that T cells in more differentiated stages, in particular in the KLRG-1^−/low^ CD57^−^ CD7^+^ intermediate and KLRG-1^+^ CD57^+^ CD7^−^ late-stage of terminal differentiation, express the TCR and bind specific tetramers with similar efficiencies; CD7^−^ T cells, however, respond less efficiently with IFN-gamma and cytolytic degranulation than the CD7^+^ KLRG-1^−/low^ CD57^−^ T cells. While hypo-responsiveness of intermediate-stage T cells is due to blockade through PD-1/PD-1L interaction, this is not the case for late-stage T cells (unpublished data). Those T cells show impaired TCR synapse formation, which is due to a selective immobility of the TCR components in the cell membrane and likely mediated by galectin-3. Our conclusion is sustained by the observation that swainsonine, which prevents galectin-3 mediated anchoring of membrane proteins by preventing branched sugar formation [15], restored the TCR synapse formation and T cell response. Hypo-responsiveness of late-stage T cells thereby seems to be due to the same mechanism as described for memory CD4^+^ and CD8^+^ T cells, not classified further in these manuscripts according to the distinct stages of T cell differentiation [15], [20], [21]. In this context it is of interest for adoptive T cell therapy that galectin-3 does not co-localize with and does not block the transgenic CAR in those T cells; CAR synapse formation occurs in both T cell subsets with same efficiencies and within the same time frame. Although late-stage T cells are hypo-responsive to TCR signals, CAR engagement of antigen mediates effector functions with equal efficiencies as in intermediate stage T cells. The CAR obviously bypasses insufficient TCR synapse formation on the membrane level through recruitment of the TCR downstream signaling leading to restoration of a functional T cell response. Experimental data from other groups help to understand our observations, since it was published that galectin-3 binds to the TCR-alpha, TCR-beta and CD3-epsilon chains, but not to the CD3-zeta chain of the TCR [22], [23]. Therefore our data strongly suggest, that a transgenic CAR, which utilizes a CD3-zeta chain for signal transduction, is not hampered by galectin-3 interaction with the TCR signal transduction machinery after antigen recognition. Life imaging moreover implies that the TCR and the CAR independently form synapses that is in contrast to the transgenic expression of a recombinant TCR that can form heterodimers with the physiological TCR [24], [25]. Even a modified transgenic TCR that does not pair with the endogenous TCR localizes in close vicinity to the endogenous TCR/CD3 in Jurkat T cells [13]. In late-stage T cells common synapses between physiological and transgenic TCR likely lead to unresponsiveness of the recombinant TCR making the redirected T cell response less effective. A single-chain chimeric antigen receptor, in contrast to the TCR, does not form common synapses with the physiological TCR although a CAR makes use of the TCR downstream signaling machinery. This may not be the case in Jurkat cells as recently reported [14]; those cells, however, exhibit altered TCR proximal signaling compared to blood T cells [26].

A significant proportion of T cells from the peripheral blood of tumor patients and from tumor lesions belong to the terminally differentiated late-stage T cell subset [16], [27]. The number of those T cells increases with progression of the disease [28]. Current improvements in adoptive cell therapy therefore aim to prevent the generation of terminally differentiated T cells by providing a short-term TCR stimulus to naive CD8^+^ T cells sufficient to induce clonal proliferation, acquisition of effector functions, and entry into the memory pool in the absence of additional stimuli [2]. Other approaches make use of pharmacological agents, like rapamycin, to improve memory formation and to expand the absolute numbers of both central and effector memory T cells [2]. These strategies may be combined with transgenic expression of a CAR that circumvents TCR hypo-responsiveness of late-stage T cells. Late-stage T cells are not only an inevitable result of repetitive antigen stimulation but also lack CCR7 [7] which has the advantage that those cells show reduced capability to re-enter lymph vessels and to re-circulate and are thereby trapped in the periphery [29] where most tumors are located.

Various physiological T cell subsets are impaired in TCR synapse formation including orally tolerized T cells [30], T cells from healthy elderly individuals [31] or T cells under chronic stimulatory conditions including rheumatoid arthritis or systemic lupus erythematosus [31]. While hypo-responsiveness in these lesional T cells is predominantly based on PD-1 - PD-L1/PD-L2 mediated repression [32], it is so far unknown whether the T cell response in those cells can also be rescued by transgenic CAR expression.

While late-stage T cells can be rescued for use in the adoptive immunotherapy, their reduced proliferative capacity and their accelerated entry into apoptosis need to be addressed. In this regard CAR-redirected T cell therapy may be combined with recently developed strategies to sustain telomerase activity, which leads to improved cytotoxic capacities, cytokine and chemokine production. Alternatively, TNF-alpha production may be inhibited, which increases the proliferative potential and telomerase activity in those cells, or transgenic CD28 may be expressed, which retards replicative senescence [33].

## Materials and Methods

### Blood samples and cell lines

Buffy coats from healthy young volunteers (n = 30) (mean 30±10 years) were obtained from the blood bank facilities of the University Hospital Cologne, Germany. Volunteers who were taking immunosuppressive drugs or who had a disease potentially affecting the immune system were excluded. Peripheral blood from 8 patients (31–74 years) with confirmed diagnosis of active CMV and lack of HIV infection was analyzed. None of the CMV patients was on therapy at the time of sampling. All blood samples were taken after patients gave their written, informed consent. This study was performed in conformity with the Declaration of Helsinki of the World Medical Association and approved by the institutional review committee of the University of Cologne (reference no 02-041). 293T cells are human embryonic kidney cells that express the SV40 large T antigen (ATCC CRL 11268). LS174T (ATCC CCL 188) is a CEA^+^ colon carcinoma cell line, and Colo320 (ATCC CCL 220.1) is a CEA^−^ cell line. OKT3 (ATCC CRL 8001) is a hybridoma cell line that produces the anti-human CD3 monoclonal antibody (mAb) (OKT3); 15E8 hybridoma cells produce the anti-human CD28 mAb (15E8) (obtained from Dr. Rene van Lier, Red Cross Bloodbank, Amsterdam, The Netherlands). All cells were cultured in RPMI 1640 medium (Invitrogen, Karlsruhe, Germany) containing 100 U/ml penicillin, 100 mg/ml streptomycin, 2 mM L-glutamine, and 10% (v/v) heat-inactivated fetal calf serum (FCS) (Invitrogen).

### Antibodies and cytokines

The following antibodies were used: APC-conjugated anti-TCR-alpha/beta(IP26) (Biolegend, Eching, Germany), PE-Cy7-conjugated anti-CD8 mAb (RPA-T8) (BD Biosciences, Heidelberg, Germany), APC-conjugated anti-CD45RO mAb (UCHL-1) (BD Biosciences), FITC-conjugated anti-CD7 mAb (4H9) (BD Biosciences), APC-conjugated anti-CD7 mAb (MEM-186) (Exbio, Eching, Germany), APC-Cy7-conjugated anti-CD45RO mAb (UCHL-1) (BD Biosciences), APC-Cy7-conjugated anti-CD3 mAb (UCHT-1) (BD Biosciences), Alexa-488-conjugated anti-KLRG-1 mAb (13A2) (kindly provided by Dr. H.P. Pircher, Freiburg, Germany), APC-Cy7-conjugated anti-CD8 mAb (SK1) (BD Biosciences), APC-conjugated anti-CD57 mAb (TB03) (Miltenyi Biotec, Bergisch Gladbach, Germany), PE-Alexa-750-conjugated anti-CD7 mAb (CD7-6B7) (Caltag, Hamburg, Germany), and PE-Cy7-conjugated anti-CD45RO mAb (UCHL-1) (BD Biosciences). To monitor intracellular galectin-3 expression T cells were stained with PE-conjugated anti-CD7 mAb (4H9) (BD Biosciences), fixed and permeabilized, and subsequentially stained with FITC-conjugated anti-galectin-3 mAb (gal-3) (Mabtech, Hamburg, Germany). Appropriate isotype antibodies (BD Biosciences) were used as controls. 15E8 and OKT3 mAbs were affinity purified from hybridoma supernatants utilizing goat anti-mouse IgG2a antibodies (Southern Biotechnology, Birmingham, Alabama, USA) immobilized on N-hydroxy-succinimid-ester-(NHS)-activated sepharose (Amersham Biosciences, Freiburg, Germany). Cells were analyzed by flow cytometry using a FACS Calibur or FACSCanto cytometer (BD Biosciences), equipped with Cell Quest or FACS DIVA software (BD Biosciences). Viable lymphocytes were gated according to forward/side scatter and lack of 7-AAD (BD Biosciences) staining. Human recombinant interleukin-2 (IL-2) and interferon-gamma (IFN-gamma) were purchased from R&D Systems (Wiesbaden, Germany). IFN-gamma in the culture medium was recorded by ELISA utilizing matched pairs of specific antibodies (clones NIB 42 and B133.5, BD Biosciences). The detection limit of the assay is 15 pg/ml IFN-gamma.

### Cell sorting

CD8^+^ CD45RO^+^ T cells were obtained from peripheral blood mononuclear cells by negative depletion magnetic cell sorting (MACS) (Miltenyi Biotec) using the “CD8^+^ T cell isolation kit” and anti-human CD45RA microbeads. CD8^+^ CD45RO^+^ T cells were separated into CD7^+^ and CD7^−^ subpopulations by using the FITC-conjugated anti-human CD7 antibody (4H9) (BD Biosciences) and anti-FITC microbeads (Miltenyi Biotec). CD8^+^ T cells were obtained from peripheral blood mononuclear cells by positive selection using anti-human CD8^+^ microbeads (Miltenyi Biotec). The purity of the isolated T cell subpopulations was routinely >95%.

### CAR mediated T cell activation

The generation of the retroviral expression cassettes for the CEA-specific CAR BW431/26scFv-Fc-CD3-zeta(#439) and retroviral transduction of T cells was described in detail [34]. Briefly, retroviral vector DNA was cotransfected with the helper plasmid DNAs pHIT60 and pCOLT (each at 1 µg DNA/10^5^ cells) into 293T cells for virus production. T cells were activated by addition of IL-2 (1,000 units/ml) and OKT3 mAb (100 ng/ml) for 48 hrs and incubated with retroviruses for additional 48 hrs. CAR expression was monitored by flow cytometry using a PE-conjugated F(ab′)_2_ anti-human IgG1 antibody (1 µg/ml) (Southern Biotechnology), which recognizes the extracellular IgG1 CH2CH3 domain of the CAR, and a FITC-conjugated anti-human CD3 mAb (UCHT-1) (BD Biosciences). After 24 hrs without stimuli redirected cytolysis was recorded by incubating T cells in increasing numbers with tumor cells (2.5×10^4^ per well) for 48 hrs in round-bottom 96-well plates, specific cytotoxicity monitored by a XTT-based colorimetric assay (Roche Applied Science, Mannheim, Germany) and the viability of tumor cells was calculated as follows:
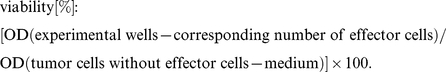



### CMV specific T cells

CD8^+^ T cells from CMV infected, HIV^−^ donors were stained for HLA-A2 with mAb (BB7.2) (BD Biosciences). Tetramer-binding T cells were detected by incubation with the PE-conjugated MHC tetrameric complexes HLA-A*0201/pp65^495–503^ (Sanquin, Amsterdam, The Netherlands). Intracellular IFN-gamma was detected in PBMC's from CMV patients stimulated for 6 hrs in presence with PE-conjugated MHC tetrameric complexes HLA-A*0201/pp65^495–503^ (Sanquin) together with CMV peptide NLVPMVATV (“CMV pp65 Peptide Mix”) or the TCR cross-linking reagent “Cytostim” (both from Miltenyi Biotec) or HIV peptide SLYNTVATL (JPT, Berlin, Germany), respectively, and stained for CD8, CD7 and CD45RO. During the final 5 hrs, brefeldin A (5 µg/ml) (BD Biosciences) was added to avoid release of cytokines from the Golgi apparatus, respectively, and cells were subsequently stained for IFN-gamma using the clone 25723.11 mAb (BD Biosciences). Alternatively, lymphocytes were activated for 6 hrs in presence of FITC-conjugated anti-CD107a mAb (H4A3) (BD Biosciences), the CMV peptide, and the agonistic anti-CD49d mAb (L25) (1 µg/ml) plus the anti-CD28 mAb (L293) (1 µg/ml) (both from BD Biosciences).

### Synapse formation

T cells (10^5^ cells) were placed on cover slips coated with poly-*L*-lysine (0.1 mg/ml) (Sigma Aldrich, Deisenhofen, Germany), the anti-CD3 mAb (UCHT-1) and the anti-CD28 mAb (CD28.2) or with the anti-CD2 mAbs (L303.1) and (L304.1) and the anti-CD28 mAb (CD28.2), or as control by an isotype-matched IgG1 (all from BD Biosciences; 10 µg/ml each). Alternatively, cover slips were coated with the BW2064/36 mAb (10 µg/ml), which is an internal image anti-idiotypic antibody, directed against the anti-CEA scFv BW431/26 [35]. Cells were activated for various time intervals (1 min until 20 min) and incubation was stopped by addition of 3.75% (w/v) paraformaldehyde. Cells were washed and blocked with 2% (v/v) human AB serum (PAA Laboratories, Coelbe, Germany) for 20 min. Synpase formation was recorded as described [36]. Briefly, cells were stained with Alexa 647- or Alexa-488-labeled cholera toxin B subunit (CTxB) (Invitrogen) for staining lipid rafts (GM1) and stained for TCR-alpha/beta(IP26) (Biolegend), CD7 (MEM-186) (Exbio), CD2 (TS1/8) (Biolegend), IgG to indicate the CAR (Medac, Hamburg, Germany), CD3 (UCHT-1) (Biolegend) and galectin-3 (M3/38) (Biolegend). Formation of synapses was indicated by accumulation of CTxB at the cell-cover slip interface as assessed by confocal laser scanning microscopy using the LSM 510 (Karl Zeiss, Oberkochen, Germany) equipped with Zen 2009 software with 630× microscope magnification. A region with accumulated TCR or CAR molecules together with an accumulation of CTxB was considered a synapse. To quantitate the recruitment of TCR and CAR to the immunological synapse, gates were automatically drawn around (i) the immunological synapse, (ii) the regions of the T cell not in contact with agonistic antibodies, and (iii) a background area. The relative recruitment index (RRI) shown in figures as CTxB intensity (relative mean fluorescence of synapse formation) was calculated as indicated: (mean fluorescence intensity (MFI) at synapse – background)/(MFI at all the cell membrane not in contact with agonistic antibodies – background). Quantitative analysis of MFI was performed automatically with the Image J programme (NIH, Maryland, USA). A minimum of 100 cells for each data point was examined quantitatively for each experiment. To block alpha-mannosidase II, T cells were incubated with swainsonine (0.5 mM) (Calbiochem, Darmstadt, Germany) for 24 hrs. Specificity of staining was assayed by incubation with the respective isotype-matched control antibody (BD Biosciences). Finally, all slides were coverslipped with Entellan (ProSciTec, Thuringowa, Australia) and analyzed by laser scan microscopy (LSM).

### Statistical analyses

Results are presented as means ± standard error of the mean (SEM). A paired and unpaired two-tailed t-test was used; p<.05 was considered significant.

## Supporting Information

Figure S1 **CMV-specific T cells in late-stage and intermediate-stage of terminal differentiation equally bind pp65 CMV tetramers.** CMV-specific CD8^+^ T cells in late or intermediate stage of terminal differentiation were identified in the peripheral blood from CMV patients with acute virus reactivation by incubation with PE-conjugated CMV peptide loaded tetramers HLA-A*0201/pp65^495–503^ and staining for CD8, CD7, CD45RO, CD57, KLRG-1, CD3 and TCR-alpha/beta as described in Materials and Methods. Cells were analyzed by flow cytometry. One representative donor out of five CMV patients is shown.
(PDF)

Figure S2 **CD7^−^ late-stage T cells are hypo-responsive to TCR/CD3 stimulation.** The CD7^−^ and CD7^+^ subsets of CD8^+^ CD45RO^+^ T cells were isolated from the peripheral blood and cultured (10^5^ cells/100 µl) with or without the agonistic anti-CD3 mAb (OKT3) (5 µg/ml), the anti-CD28 mAb (15E8) (5 µg/ml), or with IL-2 (50 U/ml). (A) IFN-gamma in the culture supernatant at day 6 was detected by ELISA. (B) To monitor CD107a^+^ IFN-gamma producing cells, cells were monitored by flow cytometry by staining with the PE-conjugated anti-IFN-gamma mAb (25723.11) and the FITC-conjugated anti-CD107a mAb (H4A3). Assays (Figs. S2A & B) were performed five times and the mean values ± SEM are shown. Statistical analyses were made using a paired t-test. * p<0.05, CD7^−^ cells compared with the corresponding CD7^+^ T cells.
(PDF)
