# Supplementary figures and images for: The CD3-Zeta Chimeric Antigen Receptor Overcomes TCR Hypo-Responsiveness of Human Terminal Late-Stage T Cells
Source: PLoS One. 2012 Jan 23;7(1):e30713. doi: 10.1371/journal.pone.0030713 (PMC3264628; doi:10.1371/journal.pone.0030713)

Figure S1

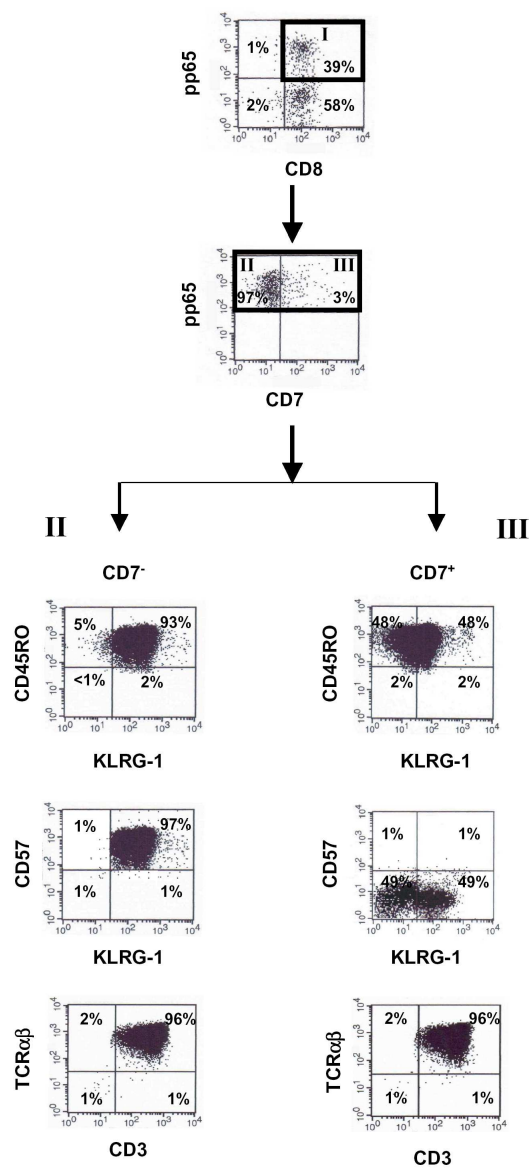

Supplement: Figure S1 — CMV-specific T cells in late-stage and intermediate-stage of terminal differentiation equally bind pp65 CMV tetramers. CMV-specific CD8+ T cells in late or intermediate stage of terminal differentiation were identified in the peripheral blood from CMV patients with acute virus reactivation by incubation with PE-conjugated CMV peptide loaded tetramers HLA-A*0201/pp65495–503 and staining for CD8, CD7, CD45RO, CD57, KLRG-1, CD3 and TCR-alpha/beta as described in Materials and Methods. Cells were analyzed by flow cytometry. One representative donor out of five CMV patients is shown. (PDF) [file pone.0030713.s001.pdf]

Figure S2

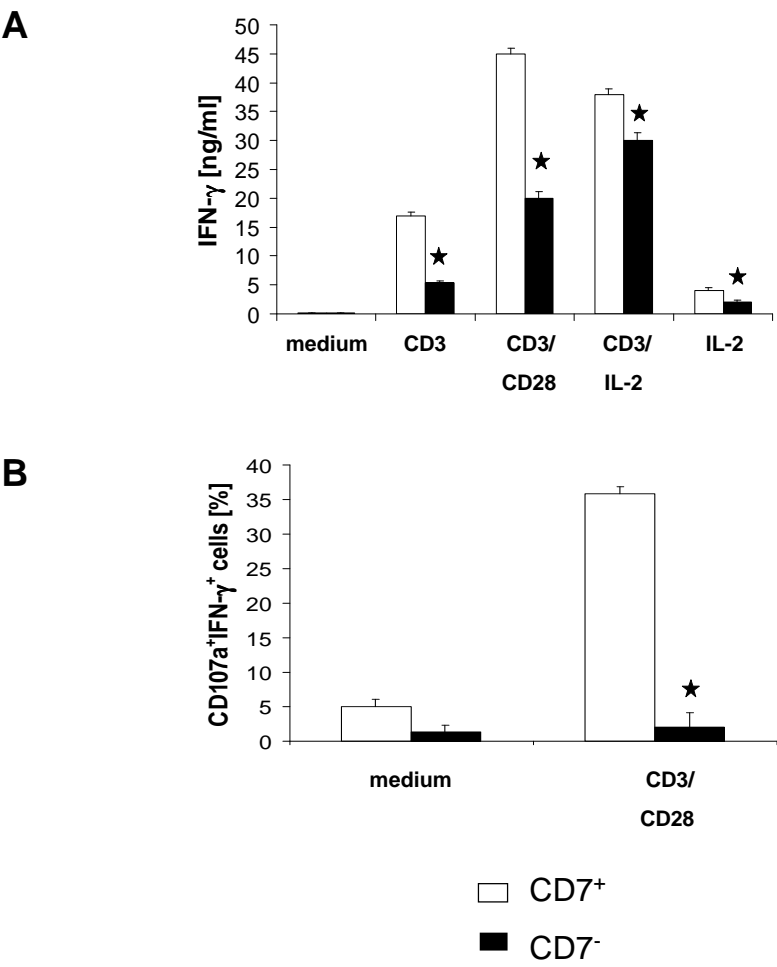

Supplement: Figure S2 — CD7− late-stage T cells are hypo-responsive to TCR/CD3 stimulation. The CD7− and CD7+ subsets of CD8+ CD45RO+ T cells were isolated from the peripheral blood and cultured (105 cells/100 µl) with or without the agonistic anti-CD3 mAb (OKT3) (5 µg/ml), the anti-CD28 mAb (15E8) (5 µg/ml), or with IL-2 (50 U/ml). (A) IFN-gamma in the culture supernatant at day 6 was detected by ELISA. (B) To monitor CD107a+ IFN-gamma producing cells, cells were monitored by flow cytometry by staining with the PE-conjugated anti-IFN-gamma mAb (25723.11) and the FITC-conjugated anti-CD107a mAb (H4A3). Assays (Figs. S2A & B) were performed five times and the mean values ± SEM are shown. Statistical analyses were made using a paired t-test. * p<0.05, CD7− cells compared with the corresponding CD7+ T cells. (PDF) [file pone.0030713.s002.pdf]
